# Combined imaging and serum biomarkers in CEUS-guided microwave ablation for hepatocellular carcinoma: A meta-analysis

**DOI:** 10.3389/fonc.2025.1687044

**Published:** 2025-11-05

**Authors:** Xiaoli Zou, Danyi Zhao, Yu Shang, Yu Song, Yimei Deng, Lin Lu

**Affiliations:** ^1^ Department of Ultrasound, Dalian Municipal Friendship Hospital, Dalian, China; ^2^ Department of Oncology, The Second Affiliated Hospital of Dalian Medical University, Dalian, China; ^3^ Department of Ultrasound, The Second Affiliated Hospital of Dalian Medical University, Dalian, China

**Keywords:** hepatocellular carcinoma (HCC), contrast-enhanced ultrasound (CEUS), microwave ablation (MWA), imaging features, serum biomarkers, meta-analysis

## Abstract

**Objective:**

This study aimed to evaluate the efficacy of a treatment strategy that actively integrates imaging features and serum biomarkers into contrast-enhanced ultrasound (CEUS)-guided microwave ablation (MWA) for hepatocellular carcinoma (HCC).

**Methods:**

A comprehensive literature search was conducted, and randomized controlled trials (RCTs) meeting the inclusion criteria were selected. The methodological quality of the included studies was assessed using the Cochrane Risk of Bias Tool, and RevMan 5.3 software was employed for meta-analysis. The primary endpoints included complete tumor ablation rate, local recurrence rate (LRR), local progression rate (LPR), recurrence-free survival (RFS), and complication rate.

**Results:**

A total of seven RCTs involving 1,039 HCC patients (407 in the treatment group, 632 in the control group) were included. Meta-analysis demonstrated the following: The complete ablation rate was significantly higher in the treatment group than in the control group (risk ratio [RR] = 1.06; 95% confidence interval [CI] = [1.01, 1.10]; *p* = 0.010). The local recurrence rate was significantly lower in the treatment group (risk difference [RD] = − 0.09; 95% CI = [− 0.17, −0.01]; *p* = 0.02). No significant differences were observed in RFS (RR = 1.11; 95% CI = [1.00, 1.24]; *p* = 0.06), LPR (RR = 1.55; 95% CI = [0.78, 3.07]; *p* = 0.21), or complication rates (RR = 1.13; 95% CI = [0.66, 1.91]; *p* = 0.66) between the two groups. Heterogeneity among studies was low (*I* ^2^ ≤ 34%), and funnel plot analysis indicated minimal publication bias.

**Conclusion:**

CEUS-guided MWA combined with imaging features and serum biomarkers is associated with significant improvements in complete tumor ablation rates and a reduction in local recurrence. This strategy provides evidence-based support for optimizing precision local control in HCC, but its impact on long-term survival requires validation through future studies with extended follow-up.

## Introduction

In the field of malignant tumor treatment, hepatocellular carcinoma (HCC), ranking as the sixth most common malignancy globally and the third leading cause of cancer-related mortality, exhibits high invasiveness and insidious onset characteristics. Consequently, most patients are diagnosed at advanced stages, missing the optimal window for surgical resection, with a long-term 5-year survival rate stagnating at 10%–15%, imposing a substantial burden on both patient quality of life and healthcare systems (1, 2). Although surgical resection remains the gold standard for radical HCC treatment, it is applicable to only approximately 20% of early-stage patients. For those with intermediate-to-advanced stages, multifocal lesions, or concomitant cirrhosis, minimally invasive therapies have emerged as critical alternatives (3, 4).

Contrast-enhanced ultrasound (CEUS)-guided microwave ablation (MWA), characterized by precise targeting, minimal invasiveness, and repeatability, has gained widespread application in HCC management. This technique induces irreversible tumor necrosis through thermal coagulation effects, achieving local radical control (5). Imaging features, serving as direct manifestations of tumor morphology and hemodynamics, can delineate lesion size, margins, internal architecture, and vascular patterns, providing essential guidance for ablation zone planning and electrode placement. Meanwhile, serum biomarkers (e.g., alpha-fetoprotein [AFP], des-gamma-carboxy prothrombin [DCP]) reflect tumor biological behavior and therapeutic response, functioning as key quantitative indicators for dynamic efficacy evaluation (6).

However, studies relying solely on imaging or serological markers have limitations: imaging features may fail to accurately identify microscopic residual lesions, whereas serum biomarkers are susceptible to interference from hepatic/renal function and other factors (7, 8). Although prior research has explored the combined application of these modalities, discrepancies in sample sizes and evaluation criteria have led to contentious conclusions. Therefore, this meta-analysis aims to evaluate the efficacy of a specific clinical strategy: the active integration of multiparametric data—specifically, quantifiable CEUS characteristics (e.g., hemodynamic perfusion patterns like “fast-in-fast-out”) and key serum biomarkers (e.g., AFP and DCP)—into the procedural planning and execution of CEUS-guided MWA for HCC. Unlike prognostic studies that merely assess correlations, our objective is to determine whether clinically acting upon this combined information leads to superior outcomes compared with a control strategy that does not formally integrate such data. This systematic review of RCTs is designed to provide the most robust evidence regarding the causal benefit of this integrated guidance strategy, and its findings hold significant clinical implications for advancing precision ablation therapy.

## Materials and methods

### Eligibility criteria

#### Inclusion criteria

##### Study design

This meta-analysis included randomized controlled trials (RCTs).

##### Participants

Eligible participants were patients with HCC confirmed either pathologically or clinically, with complete baseline data and no severe organ dysfunction.

##### Interventions

The experimental group received CEUS-guided MWA combined with imaging features and serum biomarkers. The control group received standard MWA guidance (e.g., conventional US or CEUS) without formally integrating imaging features and serum biomarkers for treatment planning and evaluation.

##### Outcomes

The primary outcomes included complete ablation rate, local recurrence rate, recurrence-free survival, local progression rate, and complication incidence. All outcomes were assessed using clearly defined evaluation methods.

#### Exclusion criteria

##### Study design

Studies that were non-RCTs, such as retrospective studies or case reports, were excluded.

##### Participants

Patients with concurrent malignancies, severe comorbidities, or incomplete data were not eligible for inclusion.

##### Data integrity

Studies with unavailable key parameters, such as procedural duration or efficacy metrics, or with ambiguous evaluation protocols, were excluded to ensure data reliability.

##### Confounding factors

Studies with uncontrolled significant confounders, such as inconsistent treatment regimens, were excluded to minimize bias.

### Search strategy

PubMed, MEDLINE, Cochrane Library, EMBASE, and Web of Science were searched for all relevant studies of interest up until July 2025 to ensure data timeliness and scientific rigor. The search strategy was designed to encompass four key concepts: (1) the disease (hepatocellular carcinoma), (2) the intervention (microwave ablation), (3) the guidance modality (contrast-enhanced ultrasound), and (4) the predictive factors (imaging features and serum biomarkers). Core concepts were linked using the Boolean operator “AND” to ensure that retrieved records pertained to the combined strategy, while synonyms and related terms within each conceptual group were combined using “OR”.

The PubMed search strategy was structured as follows: (“hepatocellular carcinoma” OR “HCC” OR “liver cancer”) AND (“microwave ablation” OR “microwave thermoablation” OR “MWA”) AND (“contrast-enhanced ultrasound” OR “contrast media” OR “CEUS” OR “ultrasonography”) AND ([“imaging features” OR “radiomic features” OR “radiomics” OR “fast-in-fast-out” OR “wash-in” OR “wash-out”] OR [“serum markers” OR “biomarkers” OR “alpha-fetoprotein” OR “AFP” OR “des-gamma-carboxy prothrombin” OR “DCP” OR “PIVKA-II”]). Similar strategies, adapted to the specific syntax of each database, were applied to the other databases. In addition, the reference lists of retrieved articles and relevant reviews were manually screened to identify any additional eligible studies.

### Study selection and data extraction

Two independent reviewers screened the titles/abstracts of all retrieved studies, excluding those that were irrelevant. Articles deemed potentially eligible underwent full-text assessment. The data extracted from each study included: (1) study characteristics (authors, publication year, country); (2) sample size and baseline data (age, gender, tumor size/number); (3) intervention details (CEUS parameters, MWA power/duration); (4) imaging features and serum biomarker evaluation (methods, cutoffs, values); and (5) outcomes (complete ablation rate, recurrence/progression rates, survival, complications). Any disagreements between reviewers were resolved through discussion or third-party adjudication.

### Quality assessment

The methodological quality of included studies was evaluated using the Cochrane Risk of Bias Tool. This assessment considered the following domains: random sequence generation, allocation concealment, blinding (participants/personnel/outcome assessors), incomplete outcome data, selective reporting, and other potential sources of bias. Assessments were independently conducted by two reviewers, and any discrepancies were resolved by consensus.

### Imaging features and serum biomarkers

Among the included studies, the most frequently utilized imaging features for planning and assessing CEUS-guided MWA were as follows. First, vascular pattern characteristics, particularly the “wash-in and wash-out” pattern, were a critical feature used across studies to define viable tumor tissue and margins. Second, tumor margin definition was considered important; poorly defined or irregular margins were often cited as an indicator for extending the ablation zone. Third, internal enhancement patterns were assessed, with heterogeneous enhancement regarded as a sign of viable tumor tissue, guiding the placement of ablation antennae.

The serum biomarkers integrated into the treatment algorithm primarily included AFP and prothrombin induced by vitamin K absence or antagonist-II (PIVKA-II; also known as DCP). AFP was the most commonly used marker. Preoperative elevation of AFP—typically > 20 or > 400 ng/mL depending on the study—was used for risk stratification. A postoperative decline, such as a reduction > 50% or normalization to < 20 ng/mL, served as a key metric for evaluating treatment response and predicting recurrence. PIVKA-II was used in several studies, with cutoff values ranging from 40 to 100 mAU/mL. Elevated preablation levels of PIVKA-II were associated with higher tumor aggressiveness and were used to justify more extensive ablation margins.

The combination of imaging features and serum biomarkers was applied dynamically during CEUS-guided MWA. Imaging defined the anatomical target, while serum biomarkers provided complementary biological information. For instance, a patient with an ill-defined margin on CEUS and a high preoperative AFP level would undergo an extended ablation protocol.

### Statistical analysis

Analyses were performed using RevMan 5.3. Dichotomous outcomes (e.g., ablation success, recurrence) were expressed as odds ratios (ORs) with 95% confidence intervals (CI), while continuous variables were analyzed using mean differences (MDs) with 95% CI. Heterogeneity was assessed via Cochran’s *Q* and *I* ^2^ tests. Fixed-effects models were applied if *p* > 0.10 and *I* ^2^ < 50%; otherwise, random-effects models were used. Subgroup/sensitivity analyses were conducted to address heterogeneity. Forest and funnel plots were employed to visualize results and assess publication bias, respectively. A *p* < 0.05 was considered statistically significant.

## Results

### Literature search results

**Figure 1:**
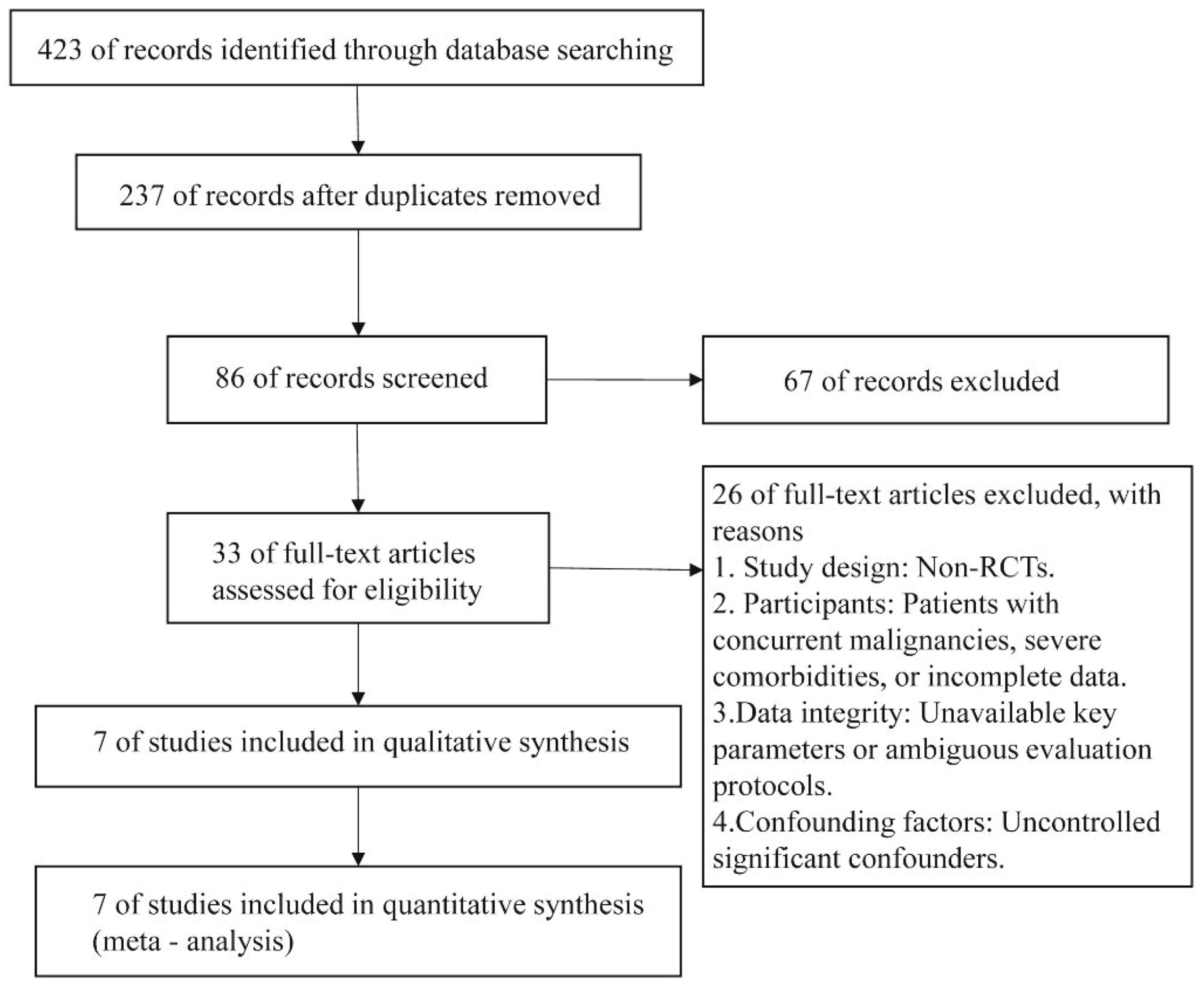
Flowchart of literature search.

The initial database searches yielded 423 articles. After applying the eligibility criteria, seven studies were included in the analysis (**Figure 1**).

### Characteristics of included studies

A total of seven studies involving 1,039 participants were included, comprising 407 cases in the treatment group and 632 cases in the control group. All studies were RCTs (**Table 1**).

**Table 1:** Baseline characteristics of included studies.

Study (year)	Treatment measures		Sample size		Primary outcomes	Randomization
	Treatment group	Control group	Treatment group	Control group		
Zhang (2025) (9)	CEUS-MWA	Standard MWA	117	128	Complete ablation rate, recurrence-free survival, and complication rate	RCT
Yan (2016) (10)	Real-time CEUS-MWA	Conventional US-MWA	50	50	Local recurrence rate	RCT
Lu MD 2005 (11)	CEUS-MWA	CEUS-RFA	49	53	Complete ablation rate, local recurrence rate, and complication rate	RCT
Desai (2025) (12)	CEUS-MWA	Conventional surgery	30	30	Local recurrence rate and complication rate	RCT
Liu (2023) (13)	CEUS-MWA	Conventional surgery	99	222	Recurrence-free survival and local progression rate	RCT
Jin (2020) (14)	CEUS-MWA	TACE+RFA	23	111	Complete ablation rate	RCT
Radosevic (2022) (15)	CEUS-MWA	CEUS-RFA	39	38	Complete ablation rate, local progression rate, and complication rate	RCT

*CEUS-MWA*, contrast-enhanced ultrasound-guided microwave ablation; *RFA*, radiofrequency ablation; *TACE*, transarterial chemoembolization; *PEI*, percutaneous ethanol injection.

### Quality assessment of included studies

**Figure 2:**
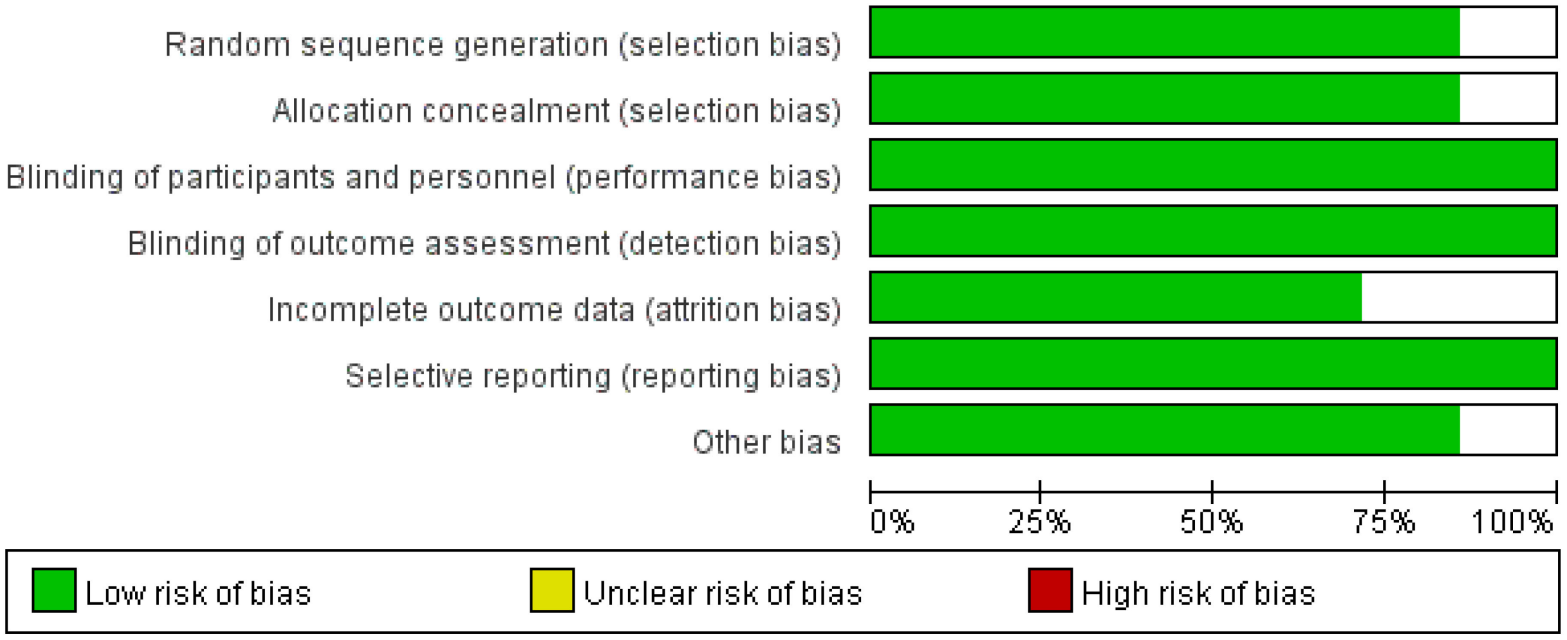
Risk of bias graph for the included studies.

All included RCTs were evaluated for quality using the Cochrane Risk of Bias Tool. Among the seven included studies, no significant sources of bias were identified, and all were rated as “low risk” (**Figure 2**).

### Meta-analysis results

#### Complete tumor ablation rate

**Figure 3:**
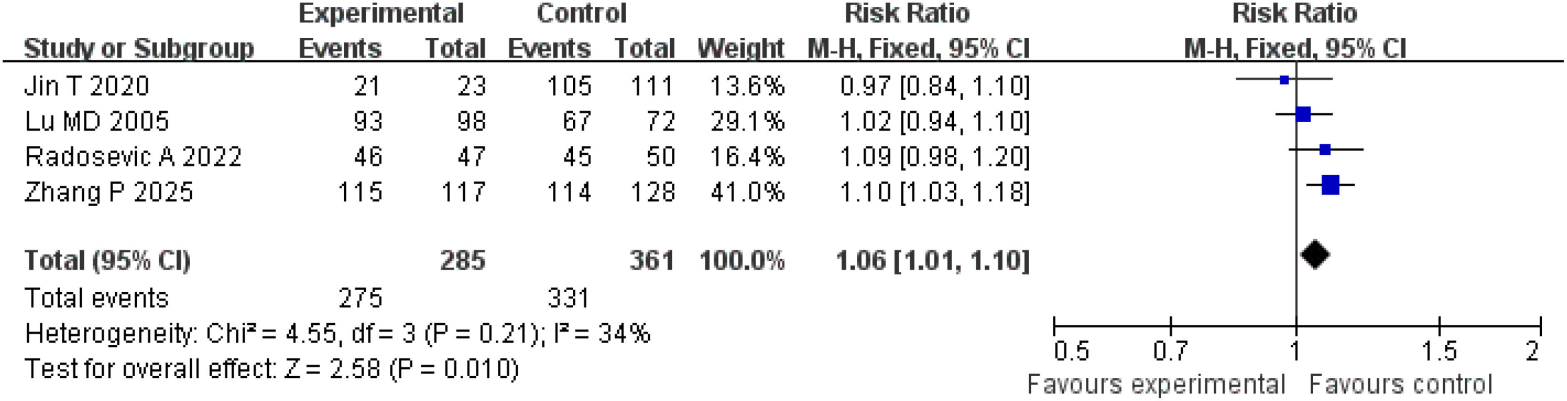
Forest plot comparing complete tumor ablation rates.

**Figure 4:**
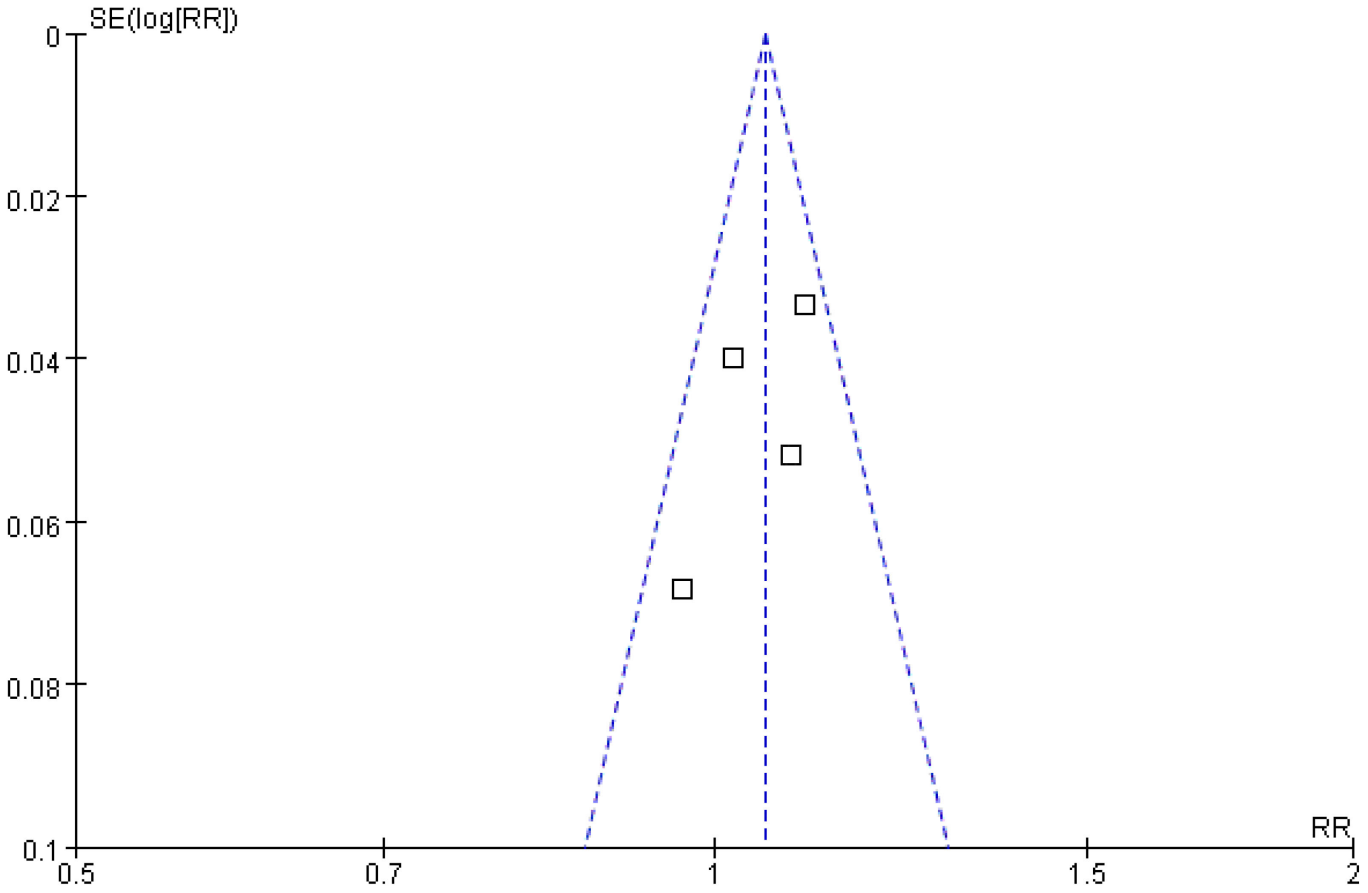
Funnel plot comparing complete tumor ablation rates.

A total of four studies were included for the analysis of the complete tumor ablation rate. The Q-test and *I* ^2^ test indicated low heterogeneity among the studies (*p* = 0.21; *χ* ^2^ = 4.55; *df* = 3; *I* ^2^ = 34%). The results demonstrated a statistically significant difference between the treatment and control groups (*Z* = 2.58; *p* = 0.010), with a pooled risk ratio (RR) of 1.06 (95% CI = [1.01, 1.10]), suggesting that the treatment group achieved superior complete tumor ablation rates compared with the control group. The funnel plot was approximately symmetric, indicating minimal publication bias (**Figures 3**, **4**).

#### Local recurrence rate

**Figure 5:**
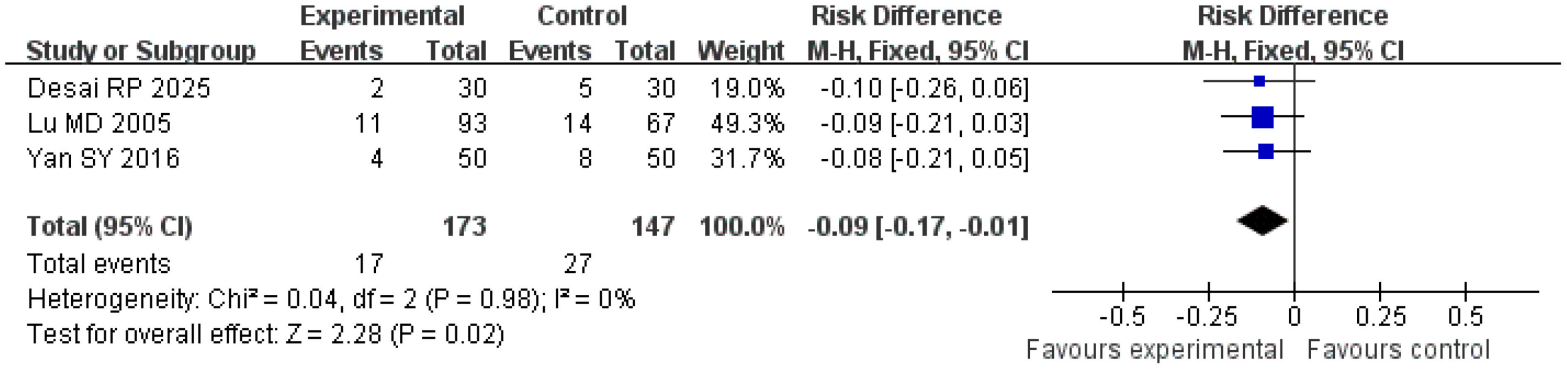
Forest plot comparing local recurrence rates.

**Figure 6:**
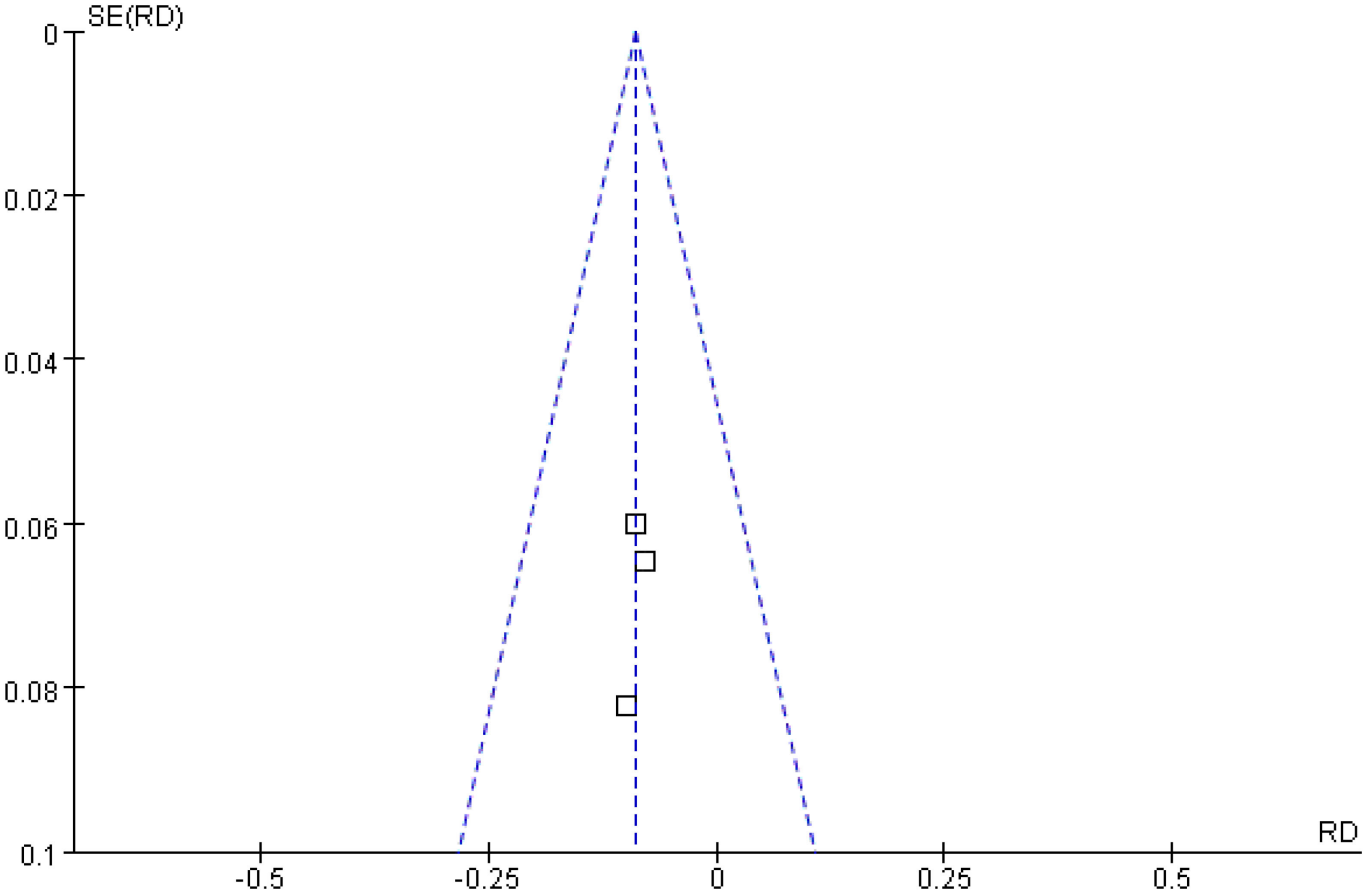
Funnel plot assessing publication bias for local recurrence rates.

A total of three studies were included in the analysis of local recurrence rates. The *Q* and *I* ^2^ test results (*p* = 0.98; *χ* ^2^ = 0.04; *df* = 2; *I* ^2^ = 0%) indicated no significant heterogeneity among the studies. Pooled effect size analysis revealed a risk difference (RD) of − 0.09 (95% CI = [− 0.17, − 0.01]), demonstrating a statistically significant difference between the two groups (*Z* = 2.28; *p* = 0.02), suggesting that the treatment group had a lower local recurrence rate compared with the control group. The funnel plot was approximately symmetric, indicating low publication bias (**Figures 5**, **6**).

#### Recurrence-free survival

**Figure 7:**
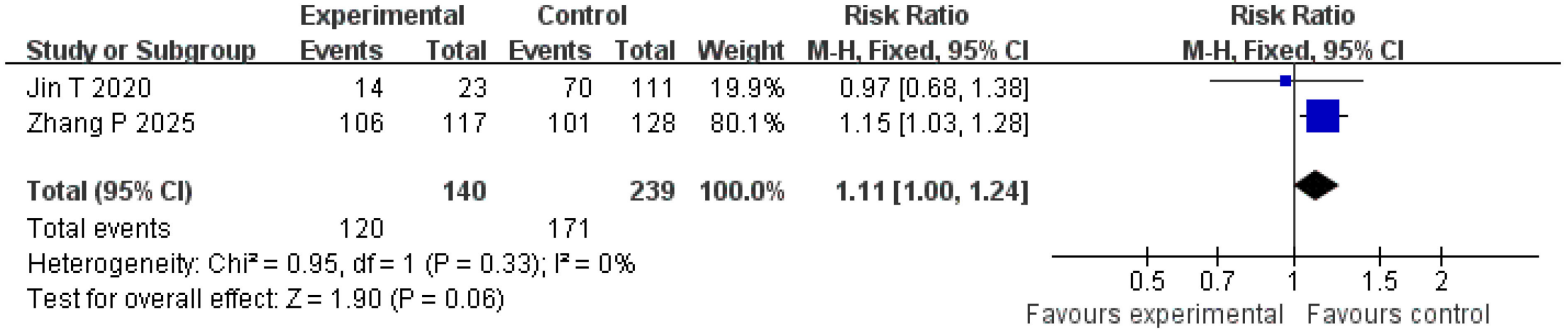
Forest plot of recurrence-free survival comparison.

**Figure 8:**
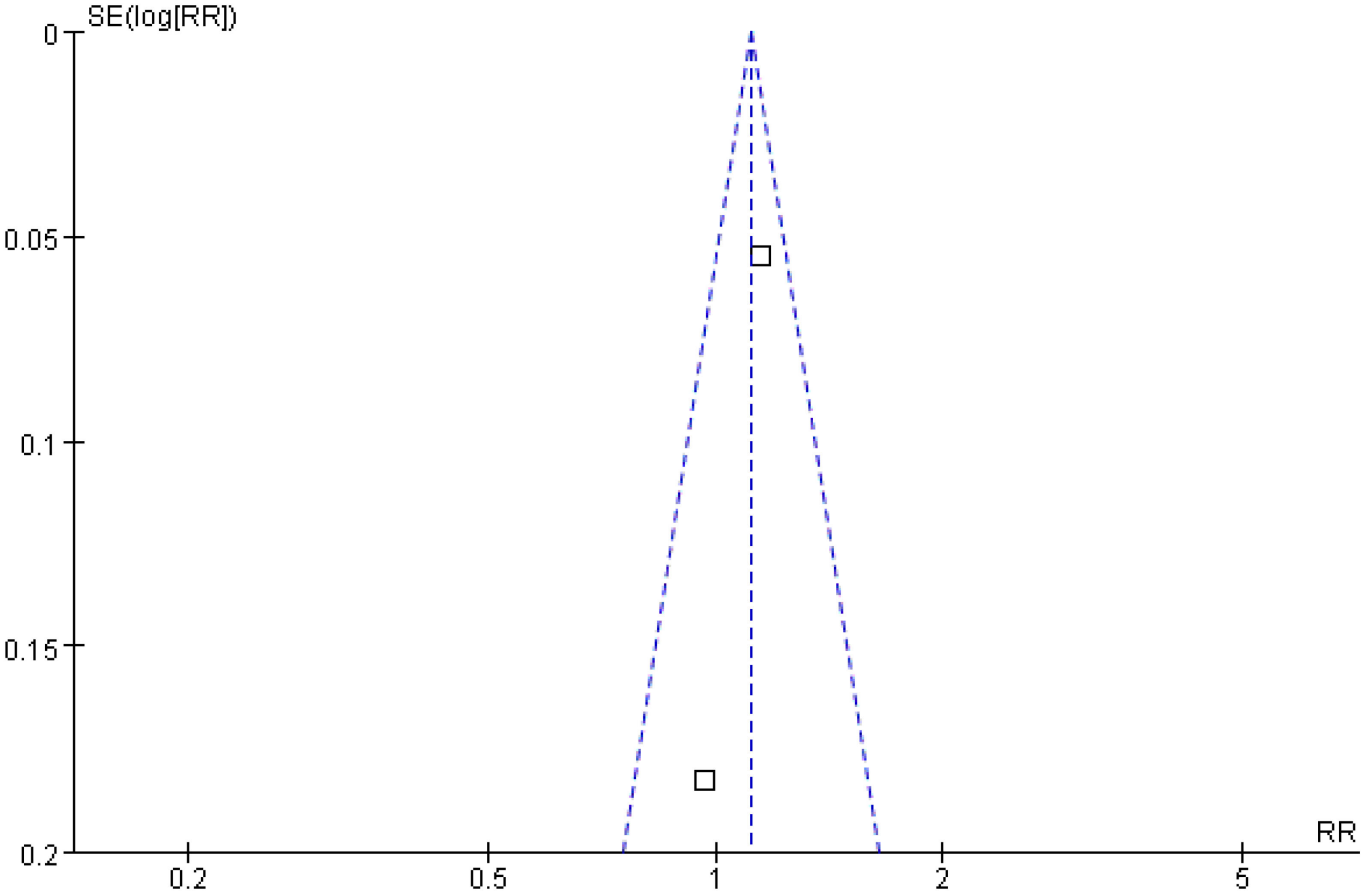
Funnel plot of recurrence-free survival comparison.

Two studies were included in the analysis of recurrence-free survival. The *Q* and *I* ^2^ test statistics revealed no significant heterogeneity among studies (*p* = 0.33; *χ* ^2^ = 0.95; *df* = 1; *I* ^2^ = 0%). Pooled effect size analysis demonstrated a RR of 1.11 (95% CI = [1.00, 1.24]), indicating a positive trend favoring the treatment group, but this did not reach conventional statistical significance (*p* = 0.06). It is important to note that this analysis, based on only two studies, is likely underpowered to detect a clinically important difference, and the nonsignificant result may reflect a type II error. The funnel plot was approximately symmetric, indicating low publication bias (**Figures 7**, **8**).

#### Local tumor progression rate

**Figure 9:**
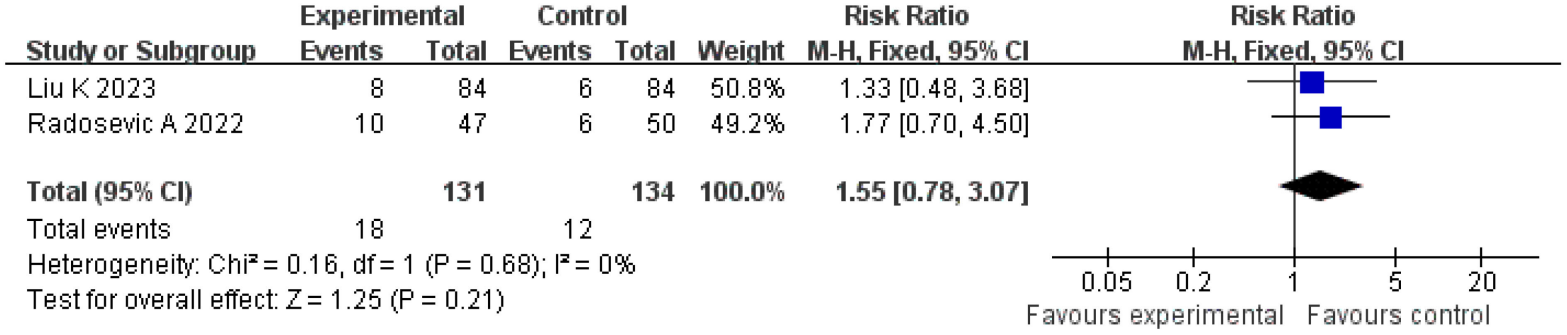
Forest plot for comparison of local progression rates.

**Figure 10:**
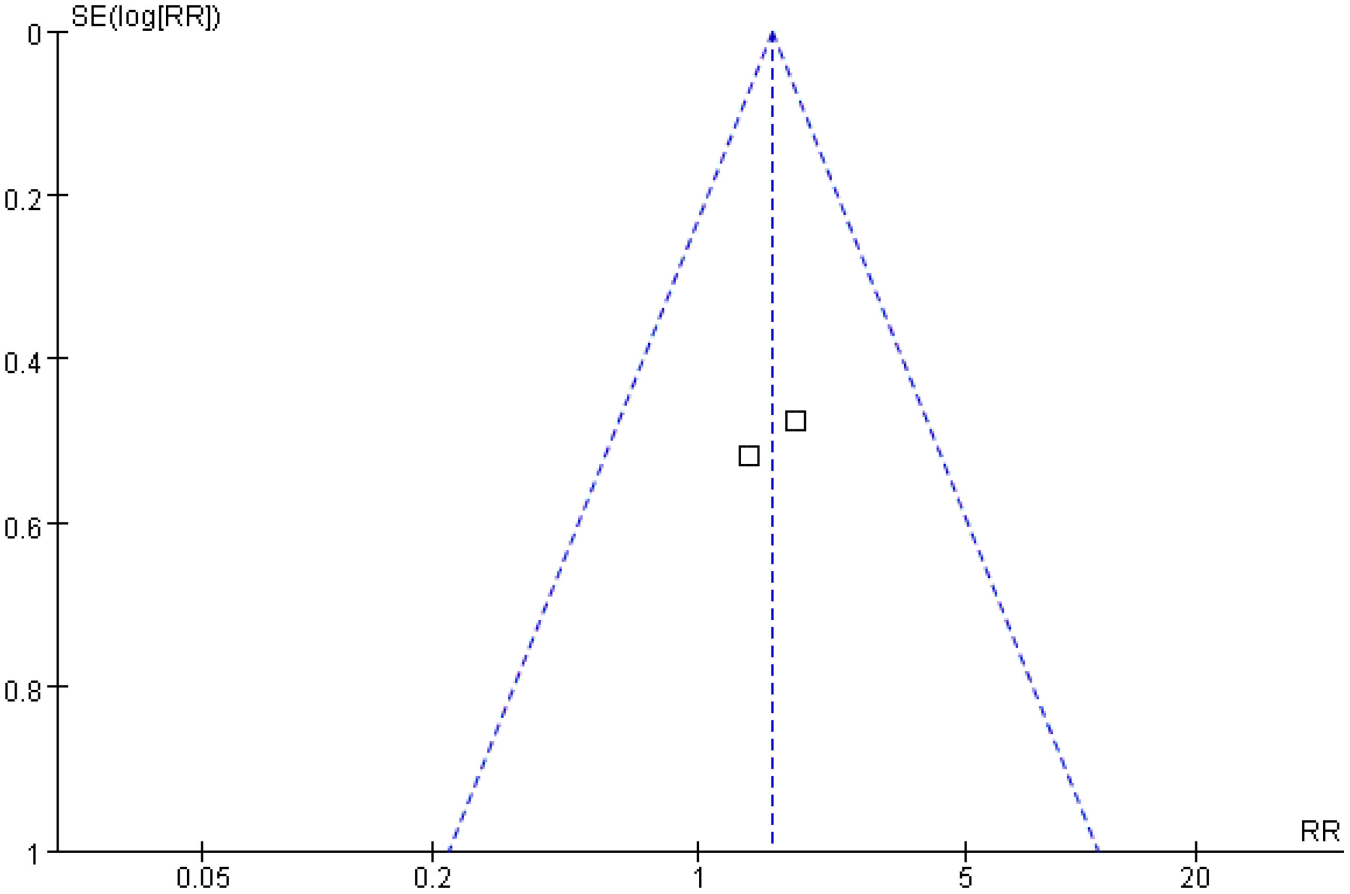
Funnel plot for comparison of local progression rates.

A total of two studies were included in the analysis of local tumor progression rates. The *Q* and *I* ^2^ test results (*p* = 0.68, *χ* ^2^ = 0.16, *df* = 1; *I* ^2^ = 0%) indicated no significant heterogeneity among the studies. Pooled effect size analysis revealed no statistically significant difference between the two groups (RR = 1.55; 95% CI = [0.78, 3.07]; *p* = 0.21). As this analysis included only two studies, the wide confidence interval, overlapping both potential harm and benefit, indicates substantial uncertainty. These results should be interpreted with caution due to the very limited power. The funnel plot was approximately symmetric, suggesting low publication bias (**Figures 9**, **10**).

### Complication incidence

**Figure 11:**
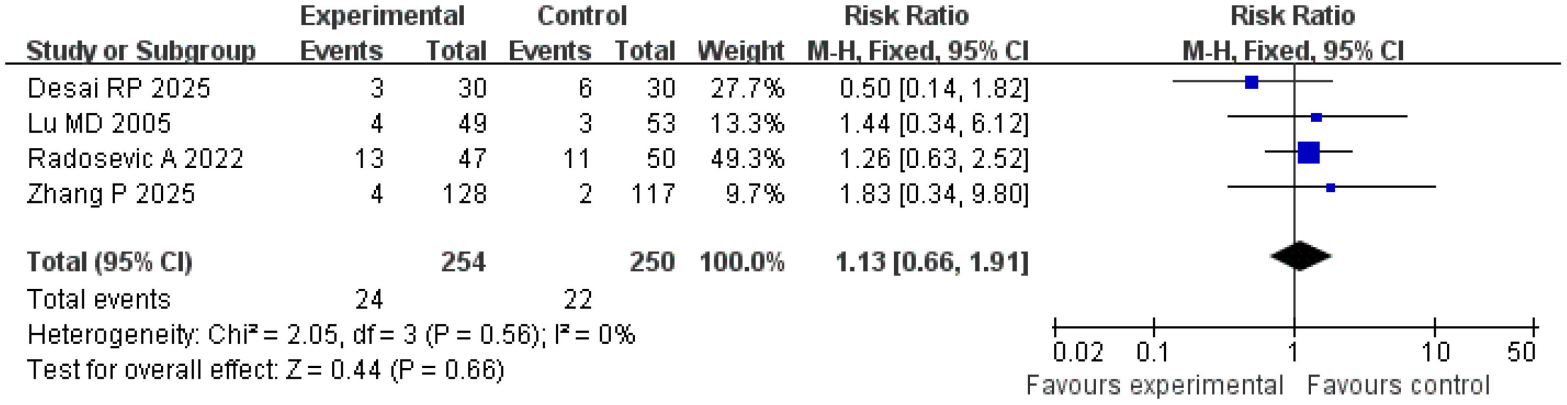
Forest plot for comparison of complication incidence rates.

**Figure 12:**
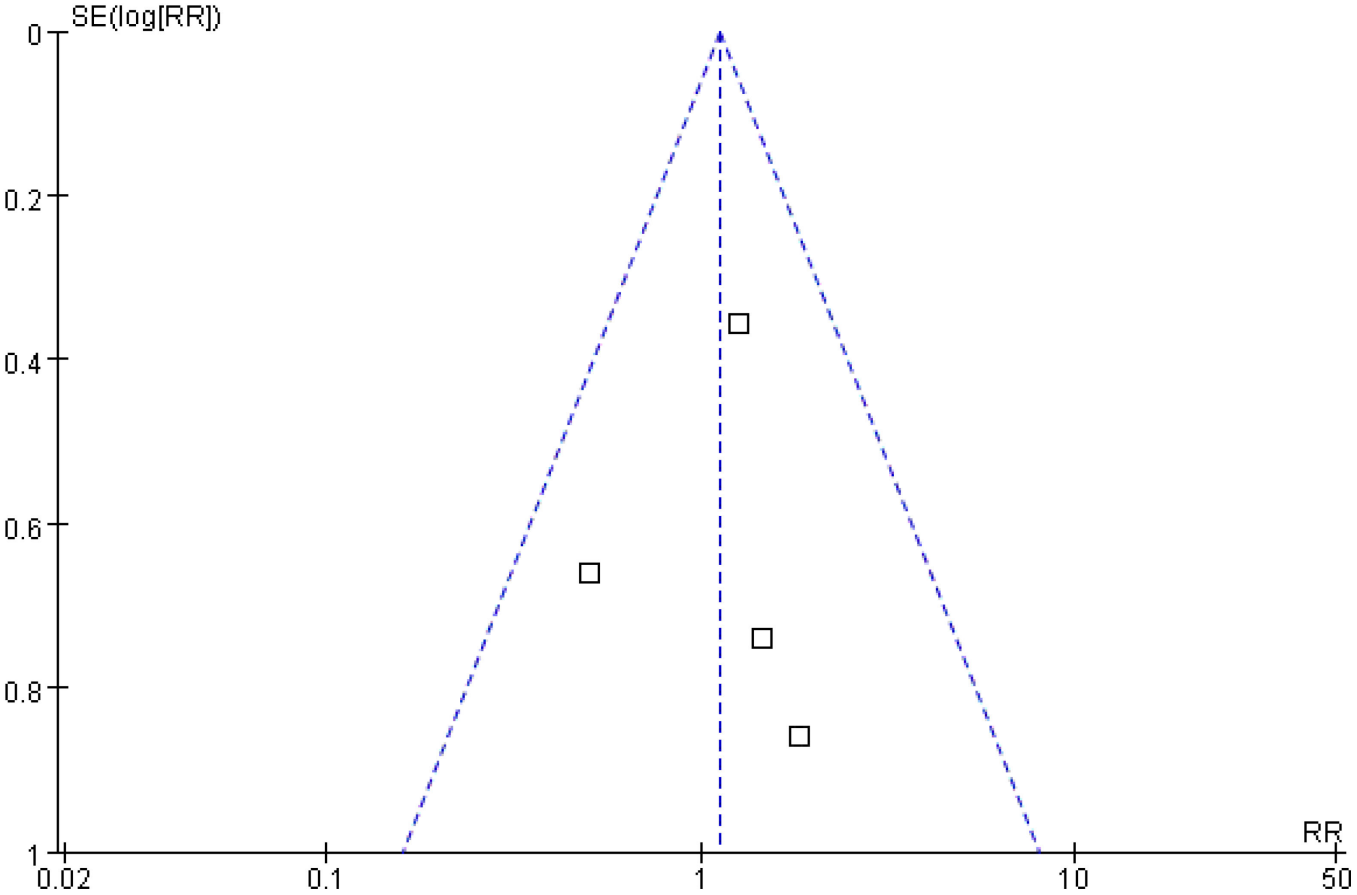
Funnel plot for comparison of complication incidence rates.

A total of four studies were included in the analysis of complication incidence. The results of the *Q* and *I* ^2^ tests were as follows: *p* = 0.56 (*χ* ^2^ = 2.05; *df* = 3), *I* ^2^ = 0%, indicating no significant heterogeneity among the studies. In the pooled effect size analysis, the RR was 1.13, with a 95% CI of [0.66, 1.91]. There was no statistically significant difference between the two groups (*Z* = 0.44; *p* = 0.66). The funnel plot was approximately symmetric, suggesting a low risk of publication bias (**Figures 11**, **12**). It is important to note that the reported complications were predominantly major adverse events. Data on minor complications (e.g., postablation syndrome, transient pain, or biochemical abnormalities) were inconsistently reported across studies, precluding their meaningful analysis.

## Discussion

HCC, a malignant tumor with high global incidence and mortality, remains a key and challenging focus in clinical research. Due to its insidious symptoms in the early stages, most patients miss the optimal timing for surgical resection at diagnosis. Therefore, minimally invasive treatments have become important options for patients with intermediate- to advanced-stage disease or those who are not eligible for surgery. CEUS-guided MWA, as a precise and minimally invasive treatment modality, induces irreversible tumor necrosis through thermal coagulation and has demonstrated significant advantages in the management of HCC. However, the traditional evaluation mode that relies solely on imaging or serological indicators has limitations. Imaging features may fail to identify small residual lesions, and serum markers can be affected by factors such as liver or kidney function. In this study, a meta-analysis was conducted to systematically evaluate the efficacy of CEUS-guided MWA combined with imaging features and serum markers in the treatment of HCC. The results showed that this strategy significantly improved the complete tumor ablation rate and reduced the local recurrence rate without increasing the risk of complications, providing important evidence-based support for the precise treatment of HCC.

The complete tumor ablation rate is the primary indicator for evaluating the efficacy of local ablation treatment, directly affecting both short-term outcomes and long-term survival of patients. This meta-analysis included four studies comprising a total of 692 patients. The results showed that the complete tumor ablation rate in the treatment group was significantly higher than in the control group (RR = 1.06; 95% CI = [1.01, 1.10]; *p* = 0.010), and heterogeneity among the studies was low (*I* ^2^ = 34%), indicating that this conclusion is highly reliable. This result may be closely related to the synergistic effect of complementary data types: real-time hemodynamic information from CEUS and quantitative biological activity from tumor markers: the real-time blood perfusion information provided by CEUS can accurately locate tumor boundaries and tiny satellite lesions, ensuring that the ablation range covers all lesions, while dynamic monitoring of serum markers (such as alpha-fetoprotein, abnormal prothrombin) can identify potential residual lesions at an early stage and guide supplementary ablation (16, 17). For example, Zhang et al. found that in patients exhibiting the “fast-in and fast-out” imaging features on contrast-enhanced ultrasound combined with a high preoperative alpha-fetoprotein level (> 400 ng/mL), the complete ablation rate was 9% higher than that in the group guided by conventional ultrasound after adjusting the ablation power and range (9). In addition, a study by Lu et al. reported that, when comparing CEUS-guided MWA and radiofrequency ablation, dynamic evaluation combined with serum markers significantly improved the complete tumor ablation rate in the CEUS-guided MWA group, further confirming the value of multimodal evaluation in optimizing the ablation strategy (11).

Local recurrence is a key factor affecting the prognosis of HCC patients, and its occurrence is closely associated with tumor residue, microvascular invasion, and incomplete treatment (18, 19). An analysis of three studies involving 320 patients in this research showed that the local recurrence rate in the treatment group was significantly lower than in the control group (RD = − 0.09; 95% CI = [− 0.17, − 0.01]; *p* = 0.02), with no obvious heterogeneity (*I* ^2^ = 0%), suggesting that combining imaging and serological indicators can effectively reduce the recurrence risk. The potential mechanism underlying this result is as follows: imaging features (such as tumor size, boundary clarity, and presence of capsule) can predict tumor invasiveness, while serum marker levels (such as alpha-fetoprotein-L3 subtype, abnormal prothrombin) can reflect tumor biological activity (20–22). For example, Yan et al. reported that in patients with a “fuzzy boundary and rich blood supply” on imaging and an abnormal prothrombin level > 40 mAU/mL, the 1-year local recurrence rate in the treatment group was 12% lower than that in the control group when ablation time was prolonged and the ablation range expanded (10). A randomized controlled trial by Desai et al. confirmed that dynamic evaluation combining contrast-enhanced ultrasound features and the postoperative decline in alpha-fetoprotein could reduce the local recurrence risk by 23%, further supporting the importance of multi-index combined monitoring in recurrence prevention and control (12).

Recurrence-free survival is an important indicator reflecting the long-term prognosis of patients. An analysis of two studies involving 379 patients in the present research showed that the recurrence-free survival in the treatment group was slightly higher than in the control group (RR = 1.11; 95% CI = [1.00, 1.24]; *p* = 0.06). Although this observed trend toward improved recurrence-free survival (RFS; RR = 1.11; *p* = 0.06) was not statistically significant, it is clinically encouraging. However, this conclusion is tentative, as the analysis was underpowered; the failure to reach statistical significance likely reflects a type II error rather than conclusive evidence of no effect. Future studies with larger sample sizes and longer follow-up are needed to definitively determine the impact on long-term survival. The observed trend may be influenced by the small sample size and differences in follow-up time: the follow-up period in the study by Liu et al. was 18 months, whereas that in the study by Zhang et al. was 24 months. Variations in follow-up periods may affect the stability of the results (9, 13). In addition, the recurrence-free survival is influenced by multiple factors, including tumor stage and liver function reserve. Differences in baseline characteristics (such as Child–Pugh grade and tumor number) in the included studies may reduce the observed significance of differences between groups (23, 24). Future studies with larger sample sizes and long-term follow-up are needed to further evaluate the impact of this strategy on recurrence-free survival.

The local progression rate reflects the invasive ability of the tumor at the primary site or in adjacent tissues. An analysis of two studies involving 265 patients in the present research showed no statistically significant difference in the local progression rate between the treatment and control groups (RR = 1.55; 95% CI = [0.78, 3.07]; *p* = 0.21). This result may be related to the multifactor-driven mechanism of local progression: pathological features, such as microvascular invasion and the distribution of satellite lesions, may exceed the predictive capacity of imaging and serum indicators, making it difficult to completely control progression risk through imaging and serological evaluations alone (25, 26). For example, a study by Radosevic et al. found that in patients with portal vein tumor thrombus, the local progression rate remained as high as 28% even when the treatment-group protocol was applied, suggesting that additional molecular markers (such as vascular endothelial growth factor, matrix metalloproteinase) may need to be incorporated to further optimize the evaluation system (15).

Safety is a key consideration in minimally invasive treatment. An analysis of four studies involving 504 patients in the present research showed no statistically significant difference in complication rates between the treatment and control groups (RR = 1.13; 95% CI = [0.66, 1.91]; *p* = 0.66), with no significant heterogeneity among the studies (*I* ^2^ = 0%), indicating that the strategy of combining imaging features and serum markers does not increase treatment risk (27). Common complications included bleeding, infection, and subcapsular liver hematoma. The occurrence of complications is closely related to the operative technique and tumor location (such as adjacent to large blood vessels or gallbladder) and is independent of the evaluation method (28). For example, Radosevic et al. confirmed that precise positioning and individualized adjustment of ablation parameters in the treatment group resulted in a severe complication rate (such as massive bleeding, bile leakage) comparable to that in the control group (< 5%), further verifying the safety of this strategy (15).

Moving forward, the logical evolution of combining imaging features and serum biomarkers lies in the development of integrated predictive algorithms. Our findings suggest that the synergistic use of anatomical and biological data provides a rich dataset suitable for machine learning or artificial intelligence models. Such tools, as explored in recent literature (29), aim to synthesize multimodal data (e.g., CEUS perfusion patterns, AFP, and DCP levels) to generate individualized prognostic scores or risk stratifications. These algorithms hold the potential to transform current standardized surveillance and follow-up protocols into a dynamic, predictive, and truly patient-specific process, ultimately enabling earlier intervention for recurrence and optimizing long-term management strategies.

This meta-analysis has several limitations. First, the relatively small number of included studies (*n* = 7), comprising a total of 1,039 patients, while demonstrating low heterogeneity, may limit the statistical power and generalizability of our findings, particularly for subgroup analyses. Second, although restricting the analysis to RCTs enhances internal validity, it excludes real-world evidence from observational studies and precludes adjustment for patient-level prognostic factors using aggregate data, potentially affecting generalizability and leaving room for residual confounding. Third, clinical heterogeneity exists across the included studies, particularly regarding control interventions (encompassing conventional ablation, surgical resection, RFA, and TACE) as well as the definitions, thresholds, and assessment timing for both imaging features and serum biomarkers. Consequently, our findings support the principle of a multimodal assessment rather than endorsing a specific, universal protocol. Although the consistent benefit observed across comparator types supports the broad utility of this strategy, this variability precludes definitive protocol recommendations. The consistent direction of benefit across studies with different control interventions strengthens the proposition that integrating imaging and biomarker data provides a universal enhancement to the CEUS-MWA procedure, regardless of the alternative treatment being used for comparison. Fourth, the relatively short follow-up durations (≤ 24 months) prevent assessment of long-term outcomes, such as overall survival and 3–5-year recurrence rates, confining our conclusions to intermediate endpoints. Fifth, safety assessment was limited by inconsistent reporting, which focused primarily on major complications and likely underestimated the burden of minor adverse events due to the absence of standardized grading systems. Finally, the conceptual amalgamation of diverse imaging and biomarker elements into a single “combined strategy” obscures the individual contribution of specific parameters, highlighting the need for future research to delineate their relative importance. Furthermore, although our search encompassed major electronic databases and clinical trial registries, we did not systematically search non-English and regional grey literature, which may have resulted in the omission of relevant studies and introduced potential selection bias.

Based on the findings and limitations of this analysis, future research should prioritize several key directions. First, large-scale, multicenter randomized controlled trials with standardized imaging protocols, harmonized biomarker thresholds, and extended follow-up durations (≥ 5 years) are needed to validate long-term survival benefits and establish durable local control. Second, studies should move beyond the current composite strategy to identify the most impactful elements through detailed analysis of specific imaging features and biomarker combinations. Third, the integration of advanced technologies—including radiomics, artificial intelligence, and emerging liquid biopsy tools such as circulating tumor DNA—should be explored to develop predictive models for treatment response and enable ultrasensitive detection of minimal residual disease. Fourth, standardized prospective collection and reporting of adverse events using validated classification systems are essential to establish a comprehensive safety profile. Finally, a future comprehensive meta-analysis incorporating well-conducted prospective cohorts could provide broader perspectives into the real-world effectiveness of this multimodal approach.

In conclusion, CEUS-guided MWA that integrates specific CEUS findings—particularly hemodynamic patterns—with serological tumor biomarkers such as AFP and DCP can significantly improve complete tumor ablation rates and reduce the local recurrence risk, offering an optimized strategy for minimally invasive treatment of HCC.

## Data Availability

The original contributions presented in the study are included in the article/supplementary material. Further inquiries can be directed to the corresponding author.
